# Vitamin D Deficiency in Saudi Patients With Rheumatoid Arthritis

**DOI:** 10.7759/cureus.34815

**Published:** 2023-02-09

**Authors:** Samar Alharbi, Razan Alharbi, Enas Alhabib, Reenad Ghunaim, Mawadah M Alreefi

**Affiliations:** 1 Department of Medicine, College of Medicine, Taibah University, Medina, SAU

**Keywords:** saudi arabia, association, disease activity, vitamin d-25 deficiency, rheumatoid arthritis

## Abstract

Background

Epidemiological studies indicate that vitamin D deficiency may increase the risk of developing autoimmune and chronic diseases such as rheumatoid arthritis (RA) and, therefore, is prevalent in patients with RA. Additionally, vitamin D insufficiency is associated with significant disease activity in patients with RA. This study aimed to assess the prevalence of vitamin D deficiency in Saudi patients with RA and determine whether there is an association between low vitamin D levels and RA disease activity.

Methodology

This cross-sectional retrospective study was conducted from October 2022 to November 2022 on patients who attended the rheumatology clinic at King Salman bin Abdulaziz Medical City, Medina, Saudi Arabia. Patients aged ≥18 years, diagnosed with RA, and not on vitamin D supplements were included. Demographic, clinical, and laboratory data were collected. Disease activity was measured using the disease activity score index of a 28-joint count using the erythrocyte sedimentation rate (DAS28-ESR).

Results

In total, 103 patients were included, with 79 patients being women (76.7%) and 24 being men (23.3%). The vitamin D level ranged from 5.13 to 94 ng/mL, with a median of 24. Of the studied cases, 42.7% had insufficient vitamin D levels, 22.3% had a deficiency, and 15.5% had severe deficiency. There were statistically significant correlations between the median vitamin D level and C-reactive protein (CRP), the number of swollen joints, and DAS. A lower median vitamin D level was detected among cases with positive CRP, swollen joints >5, and higher disease activity.

Conclusions

Patients with RA in Saudi Arabia were more likely to have low vitamin D levels. Moreover, vitamin D deficiency was linked to disease activity. Therefore, the measurement of vitamin D levels in patients with RA is essential, and vitamin D supplements might be important to improve disease outcomes and prognoses.

## Introduction

Vitamin D plays a role in maintaining a healthy mineralized skeleton by influencing the metabolism of calcium and phosphate [1]. Vitamin D is a secosteroid, a compound similar to a steroid but without two of the four carbon atoms in the steroid B ring [2,3]. In recent years, cells in the immune system have been found to contain the vitamin D receptor (VDR), and the fact that several of these cells release vitamin D has raised the possibility of its immunoregulatory qualities [4]. In addition, in the 1980s, researchers noticed that some tissues, other than the kidneys, can produce the active form of vitamin D, which is not regulated by parathyroid hormone (PTH), and identified high-VDR expression in these tissues [5]. The active form of vitamin D modulates the immune system and reduces the activation of the acquired immune system [6]. Vitamin D deficiency has been associated with altered cellular functions and increased risk of inflammatory diseases, such as rheumatoid arthritis (RA) [7]. Several studies have found a relationship between vitamin D deficiency and autoimmune diseases, such as type 1 diabetes mellitus (T1DM) [8], multiple sclerosis (MS) [9], systemic lupus erythematosus (SLE) [10], and RA [11]. However, whether vitamin D deficiency is associated with the pathogenesis of autoimmune diseases or affects the severity and progression of the disease remains unclear [12]. Vitamin D deficiency is common in patients with RA and is associated with disease activity [13,14]. In addition, according to RA-related studies, vitamin D could be a potential therapeutic biomarker and can be used to monitor disease progression as well as treatment efficacy in patients with RA [15].

This study investigated the prevalence of low-serum vitamin D levels in Saudi patients with RA and determined the impact of low-serum vitamin D levels on RA disease activity.

## Materials and methods

Study design and study population

A cross-sectional study was conducted using medical records of patients with RA who visited the rheumatology clinic at King Salman bin Abdulaziz Medical City, Medina, Saudi Arabia, from October 2022 to November 2022. The inclusion criteria were patients aged 18 years or older who were diagnosed with RA. The diagnosis was made according to the 2010 RA classification criteria. Patients younger than 18 years of age and on vitamin D supplements were excluded. In total, 103 patients were included in this study.

Data collection

The following data were collected: sociodemographic information (age and sex) and disease-related variables, including disease duration, 28-item tender joint count (TJC28), 28-item swollen joint count (SJC28), and RA-specific treatment, including glucocorticoids, disease-modifying antirheumatic drugs, and biologic therapy. Baseline laboratory investigations to monitor the RA profile as part of routine follow-up care for the patient, including C-reactive protein (CRP) and erythrocyte sedimentation rate (ESR), were also documented. The rheumatoid factor (RF) and anticyclic citrullinated peptide antibody (anti-CCP) status were also documented. Serum vitamin D levels were measured using 25-hydroxyvitamin D (25(OH)D) total commercial radioimmunoassay in a single laboratory. Vitamin D insufficiency, deficiency, and severe deficiency were defined as a serum level of 20-30, <20, and <12 ng/mL, respectively.

Disease activity was defined based on the disease activity score index of a 28-joint count (DAS28) using the ESR. Patients were categorized into groups according to DAS28: remission (<2.6), low disease activity (2.6-3.2), medium disease activity (3.2-5.1), and high disease activity (>5.1).

Ethical and administrative considerations

Ethical approval was obtained from the Scientific Research Ethics Committee of the College of Medicine, Taibah University, project ID TU-004-22. All the enrolled subjects signed a consent form before participating in the study.

Statistical analysis

Data analysis was performed using SPSS version 22 (IBM Corp., Armonk, NY, USA), with qualitative data presented as numbers and percentages. Quantitative data tested for normality by the Kolmogorov-Smirnov test were described as mean and standard deviation for normally distributed data as well as median and range for nonnormally distributed data. The appropriate statistical test was applied according to data type: chi-square test for categorical variables, student’s t-test for continuous variables, and Pearson and Spearman correlation to correlate continuous variables.

## Results

This study included 103 Saudi patients with RA, with a mean age (± standard deviation, or SD) of 51.06 ± 15.58 years, comprising 79 women (76.7%) and 24 men (23.3%). Disease duration (measured in years) was 1 to 5, 5 to ≤10, >10, and less than one year in 60.2%, 25.2%, 9.7%, and 4.9% of patients, respectively. RF was positive in 63.1%, and anti-CCP was positive in 40.9%. The baseline characteristics of the patients are summarized in Table 1.

**Table 1:** Sociodemographic characteristics of the studied cases. SD, standard deviation; CCP, cyclic citrullinated peptide; RF, rheumatoid factor

	*n* (*N *= 103)	Percentage (%)
Age (years)		
Mean ± SD (Range)	51.06 ± 15.58 (18-85)	
Median	51 (18-85)	
Gender		
Female	79	76.7
Male	24	23.3
Disease duration (years)		
<1	5	4.9
1-5	62	60.2
>5 to ≤10	26	25.2
>10	10	9.7
Anti-CCP		
Negative	55	59.2
Positive	38	40.9
RF		
Negative	38	36.9
Positive	67	63.1

Regarding RA medications, 65%, 61.2%, and 57.3% of the patients were on methotrexate, hydroxychloroquine, and prednisone, respectively, at the time of vitamin D measurement, as shown in Table 2. 

**Table 2:** Distribution of drug use in the studied cases NSAID, nonsteroidal anti-inflammatory drug

	*n* (*N *= 103)	Percentage (%)
Actemra	2	1.9
Rituximab	1	1.0
Abatacept	1	1.0
Methotrexate	67	65.0
Adalimumab	17	16.5
Denosumab	4	3.9
Etanercept	6	5.8
Tofacitinib	4	3.9
Prednisone	59	57.3
Leflunomide	13	12.6
NSAID	21	20.4
Hydroxychloroquine	63	61.2
Sulfasalazine	8	7.8

Regarding clinical features, 58.3% of the patients had no tender joints, while 16.5%, 15.5%, and 9.7% of the patients had ≤5, 6 to 10, and more than 10 tender joints, respectively. The SJC was ≤5 and >5 in 11.7% and 6.8% of the patients, respectively. ESR was high in 57.3%, and CRP was positive in 39.8% of the patients. The median DAS28 was 2.65 (range 0-5), based on which, 47.6% of the patients were in remission, while 27.2%, 19.4%, and 5.8% of the patients had moderate disease activity, low disease activity, and high-disease activity, respectively. Clinical and laboratory characteristics at the time of vitamin D measurement are summarized in Table 3.

**Table 3:** Laboratory and clinical findings of the studied cases. ESR, erythrocyte sedimentation rate; CRP, C-reactive protein; TJC, tender joint count; SJC, swollen joint count; DAS28, disease activity score 28

	*n* (*N* = 103)	Percentage (%)
ESR		
Normal	44	42.7
Abnormal	59	57.3
CRP		
Negative	62	60.2
Positive	41	39.8
TJC		
0	60	58.3
≤5	17	16.5
6–10	16	15.5
>10	10	9.7
SJC		
0	84	81.6
≤5	12	11.7
>5	7	6.8
DAS28		
<2.6	49	47.6
2.6-3.2	20	19.4
>3.2 to 5.1	28	27.2
>5.1	6	5.8

The median vitamin D level was 24 (range 5.13-94). Of the studied cases, 42.7% were vitamin D insufficient, while 22.3% and 15.5% of the patients had vitamin D deficiency and severe deficiency, respectively, as shown in Table 4.

**Table 4:** Prevalence of vitamin D deficiency among studied patients.

Vitamin D	*n* (*N* = 103)	Percentage (%)
Severe deficiency: <12 ng/mL	16	15.5
Deficiency: <20 ng/mL	23	22.3
Insufficient: 20-29 ng/mL	44	42.7
Normal: 30-50 ng/mL	20	19.4

There was no correlation between vitamin D deficiency and the type of treatment for RA. We added the table to the results (Table 5).

**Table 5:** Correlation between RA treatment type and vitamin D deficiency. MC, Monte Carlo; NSAID, nonsteroidal anti-inflammatory drug

	Vitamin D deficiency					P-value
	Severe deficiency n = 16 (%)	Deficiency n = 23 (%)	Insufficient n = 44 (%)	Normal n = 20 (%)	MC	
Methotrexate	13 (81.2)	15 (65.2)	26 (59.1)	13 (65.0)	2.54	0.469
Adalimumab	2 (12.5)	7 (30.4)	8 (18.2)	0	7.47	0.058
Denosumab	1 (6.2)	1 (4.3)	2 (4.5)	0	1.11	0.77
Etanercept	2 (12.5)	3 (13)	1 (2.3)	0	5.73	0.125
Tofacitinib	1 (6.2)	2 (8.7)	0	1 (5)	3.51	0.319
prednisone	8 (50)	15 (65.2)	24 (54.5)	12 (60)	1.13	0.769
Vitamin D3	1 (6.2)	5 (21.7)	10 (22.7)	0	7.13	0.068
Leflunomide	4 (25)	2 (8.7)	4 (9.1)	3 (15)	3.14	0.370
NSAID	3 (18.8)	4 (17.4)	10 (22.7)	4 (20)	0.304	0.959
hydroxychloroquine	8 (50)	15 (65.2)	27 (61.4)	13 (65)	1.12	0.771
Sulfasalazine	0	3 (13)	3 (6.8)	2 (10)	2.44	0.487

Statistically significant correlations between median vitamin D level and CRP, number of swollen joints, and DAS28 were observed. Lower median vitamin D was detected in patients with positive CRP, more than five swollen joints, and higher disease activity by DAS28 (Table 6).

**Table 6:** Correlation of clinical and laboratory characteristics with vitamin D levels. ^*^Z, Mann-Whitney U test, and KW, Kruskal-Wallis test. CRP, C-reactive protein; DAS28, disease activity score index 28; TJC, tender joint count; SJC, swollen joint count; CCP, cyclic citrullinated peptide; RF, rheumatoid factor

	Vitamin D, median (range)	Test of significance
Gender		
Female	22 (5.13-94)	*Z *= 1.14, *P* = 0.253
Male	26.7 (5.19-75)	
Duration (years)		
<1	24 (8.7-50)	KW = 4.33, *P *= 0.228
1-5	23 (5.19-94)	
>5 to ≤10	22.5 (5.13-75)	
>10	29.5 (15.18-39)	
Anti-CCP		
Negative	22 (5.13-45.8)	*Z *= 1.47, *P *= 0.142
Positive	25.5 (5.19-94)	
RF		
Negative	24 (5.4-40)	*Z *= 0.246, *P *= 0.806
Positive	23 (5.13-94)	
CRP		
Negative	26.72 (5.13-94)	*Z* = 2.75, *P* = 0.006*
Positive	20.6 (6.2-41.69)	
TJC		
0	24.5 (5.4-94)	KW = 3.53, *P* = 0.316
≤5	27 (5.13-33)	
6–10	21 (5.19-45.8)	
>10	18(8.2-34)	
SJC		
0	24.5 (5.4-94)	KW = 8.14, *P* = 0.017*
≤5	20.09 (5.13-45.8)	
>5	11.07 (5.19-27.89)	
DAS28		
<2.6	26 (5.4-94)	KW = 9.88, *P* = 0.02*
2.6-3.2	24.18 (6.2-41.69)	
>3.2 to 5.1	21 (5.13-45.8)	
>5.1	21 (5.13-45.8)	

**Figure 1:**
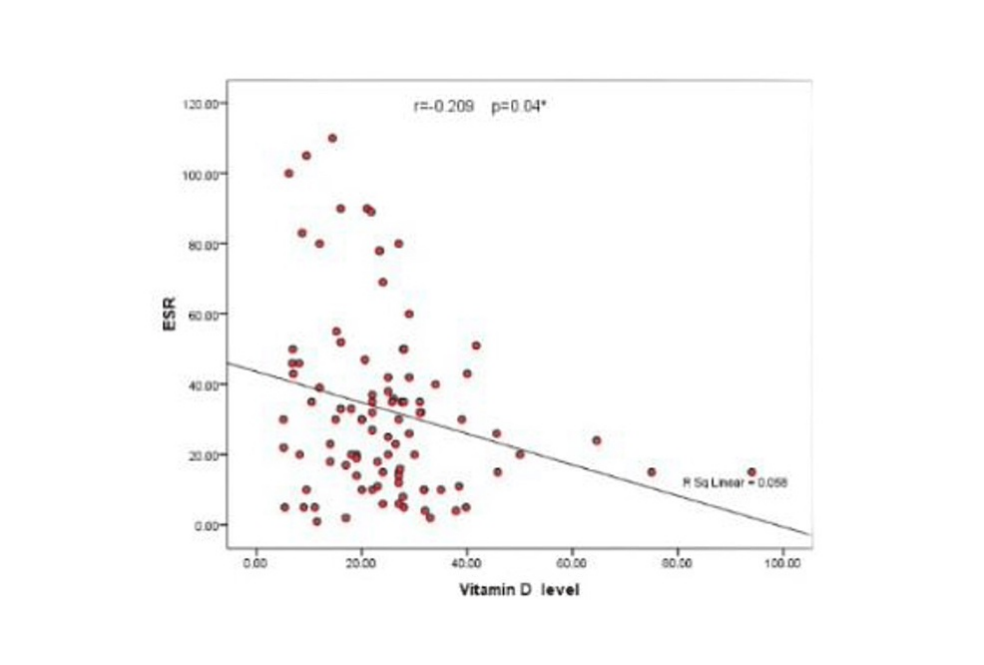
Scatter diagram showing the correlation between vitamin D levels and ESR. ESR, erythrocyte sedimentation rate

**Figure 2:**
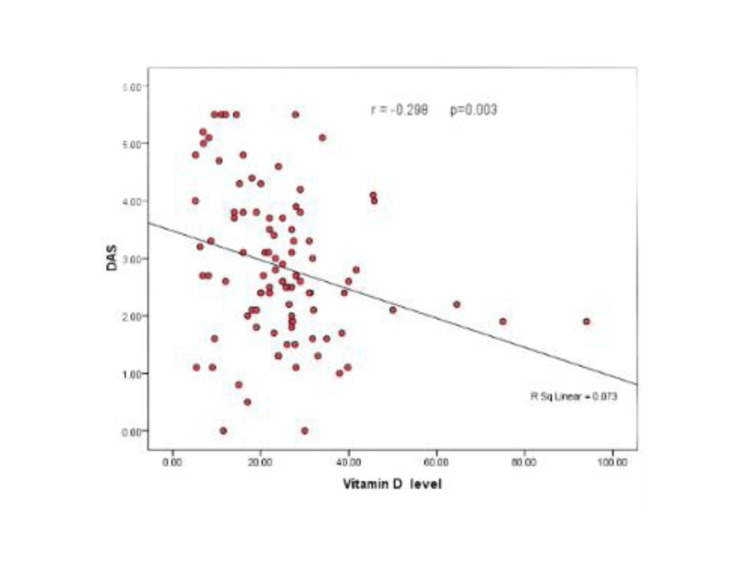
Scatter diagram showing the correlation between vitamin D levels and DAS. DAS, disease activity score index

In addition, a statistically significant negative correlation between vitamin D and ESR levels as well as DAS28 was detected, as shown in Table 7, Figure 1, and Figure 2.

**Table 7:** Correlation of vitamin D level with ESR and DAS28. *r *represents the Spearman correlation coefficient. ^*^Statistically significant. ESR, erythrocyte sedimentation rate; DAS28, disease activity score 28

	Vitamin D	
	r	P
ESR	-0.209	0.04*
DAS28	-0.298	0.003*

## Discussion

RA is a chronic inflammatory and autoimmune disease that causes synovitis of the peripheral joints and extra-articular symptoms. Uncontrolled inflammation can lead to joint damage, loss of function, and disability if left untreated. RA is caused by both genetic and nongenetic factors, such as hormonal, environmental, and infectious factors. Vitamin D could be an environmental factor, and immunological research has indicated that many chronic and immune disorders are caused by vitamin D deficiency [13,16]. It has been shown that vitamin D affects the innate and adaptive immune systems primarily via toll-like receptors (TLRs) and differentiation of T-cells, predominately Th17 cells, which play an essential role in the pathogenesis of RA [17].

Vitamin D deficiency is common in patients with RA, with a prevalence estimated to range from 30% to 63% [18]. Our study found that low vitamin D levels were common in patients with RA, among which, 42.7% were vitamin D-insufficient, while 22.3% and 15.5% had vitamin D deficiency and severe deficiency, respectively. Several studies have shown that patients with RA commonly have low vitamin D levels. In a previous study conducted in Italy, 1,191 consecutive patients with RA (85% were women) from 22 Italian rheumatology facilities and 1,019 controls who did not take vitamin D supplements were compared. The results showed that 55% of the patients with RA did not use vitamin D supplements and 52% of them had vitamin D deficiency (25(OH)D levels <20 ng/mL), which is slightly higher than our findings [18]. Another Saudi cross-sectional study included a sample of 102 patients with RA, aged between 22 and 75 years, from a population of 239 patients with RA of both genders. In the study sample, vitamin D deficiency was found in 59 patients (57.8%; 25(OH)D value 20 ng/mL), which is higher when compared to our study’s results. Furthermore, vitamin D insufficiency was found in 32 patients (31.4%; 25(OH)D 20-30 ng/mL) [19], which is lower compared to our results.

Although most previous studies have reported similar findings, some case-control studies failed to find differences in vitamin D status between patients with RA and controls [20,21].

In our study, we also observed a positive association between low vitamin D levels and disease activity in patients with RA. An inverse relationship between vitamin D levels and disease activity has been reported in several previous studies [13,14,16,18]. Similar findings were obtained in Saudi Arabia, where there was a significant negative correlation between vitamin D and disease activity in patients [2,19,22]. Although most previous studies observed an inverse correlation between vitamin D deficiency and RA disease activity, some studies did not show a strong association between vitamin D level and RA disease activity [20,23,24]. Blaney et al. presumed that vitamin D could be used as a clinical biomarker for RA and other autoimmune diseases [25]. A previous Saudi study found that low vitamin D levels in patients with RA could be a reliable indicator of high disease activity. Low vitamin D has been defined as a level less than 12.3 ng/mL [19]. Additionally, it has been shown that vitamin D intake negatively correlates with RA [26].

Limitations

The limitations of this study include its single center and small sample size, as well as its design, which includes the drawbacks common to cross-sectional studies, such as an unknowable temporal sequence of linked factors. The lack of a control group is also a limitation. Furthermore, confounding variables such as sun exposure, food habits, and nutritional status must be considered. Further prospective, multicenter, and large-sample-size studies are required.

## Conclusions

Our results show that vitamin D deficiency was common in Saudi patients with RA. Additionally, vitamin D deficiency was correlated with RA disease activity. Therefore, the measurement of vitamin D levels in patients with RA is essential, and vitamin D supplements might be important to improve disease outcomes and prognoses. Further prospective trials are required to assess the efficacy of vitamin D supplementation in the treatment and amelioration of RA symptoms and inflammatory responses. Additionally, the optimal vitamin D dose and intervention duration must also be determined.
